# Supplementary material for: Effects of water, energy, and food security on household well-being
Source: PLoS One. 2024 Jul 11;19(7):e0307017. doi: 10.1371/journal.pone.0307017 (PMC11238993; doi:10.1371/journal.pone.0307017)
Supplement: Attachment — Submitted filename: Response to Reviewers Comments-Updated (2) (2).docx [file pone.0307017.s002.docx]

**AUTHORS’ RESPONSE TO REVIEWERS' COMMENTS**

We appreciate the editors’ and the reviewers' constructive comments that have further improved this manuscript. Based on your comments and other suggestions made, we provide the following responses.

**Reviewers' comments**

**General comment**

Please work on the editing and proof-reading of the manuscript and ensure you follow the style of referencing for the journal. Please ensure all references are cited appropriately.

Response. We appreciate the reviewer's input. We have thoroughly read the manuscript and performed all necessary editing and proofreading. Again, we have cross-checked all the references cited in the text and followed the journal referencing style.

**Section 2**

**Comment #1:** These have been provided. However, some editorials need to be done on lines 229 and 210.

Response: We thank the reviewers for pointing this out. We have edited these lines accordingly.

**Section 3**

**Comment #2:** The reference provided by the authors is inaccurate and this cannot be verified.

Response: We thank the reviewers for this comment. We have updated the references (see line 257).

**Comment #5:** I am not comfortable with the response to this query. Readers will be interested to know the meaning of resource security and the contexts in which it is used in the write-up.

Response: We thank the reviewers for this comment. The term resource security was used to denote water security, energy security, and food security but not as a composite index. However, for the purpose of clarity, we have replaced the term "resource security" in the study with "water, energy, and food security" in the text where appropriate.

**Section 4**

**Comment #2:** This has been addressed. However, the reference lines provided by the authors are misleading.

Response: We thank the reviewers for pointing this out. We have updated the reference in the study accordingly.
