# Supplementary material for: Effects of water, energy, and food security on household well-being
Source: PLoS One. 2024 Jul 11;19(7):e0307017. doi: 10.1371/journal.pone.0307017 (PMC11238993; doi:10.1371/journal.pone.0307017)
Supplement: Attachment — Submitted filename: Comments to the authors.docx [file pone.0307017.s003.docx]

**Comments to the authors**

**In a positive note, the authors have raised an important issue of concern in Africa, well-being affected by energy, food, and water security. These are issues of huge policy relevance. At its weakest, however, the article suffers from measurement and estimation problems as shown below.**

1. WMS (Water supply systems) can indeed vary by household, even within the same locality. Factors such as infrastructure condition, household connections, and water usage patterns can lead to variations in water supply. However, in one village, water could be centrally managed, making this variable to be rather fixed for all households residing in that village. To deal with the fixed effects nature of the variable in a village where several sample households might have been considered for the analysis, there is a need to employ such statistical techniques as village fixed-effects regression to account for village-specific differences while estimating the effects of water management on water security.
2. Remittance and employment status can affect water security in multiple ways. Remittances can increase a household’s purchasing power, enabling them to invest in better water infrastructure or access alternative water sources. Similarly, employment status can influence water security as it affects a household’s income stability and ability to afford reliable water services. Nevertheless, the authors disregard this reality in their estimation.
3. Regarding livestock ownership (LSO), it is true that households with more livestock may have less water available for other purposes. This is because livestock require water for their own needs, and households often share their water supply with the animals. As a result, the water that could have been used for domestic or other productive purposes is allocated to livestock, potentially affecting overall water availability for the household. However, this is not considered in the regression model used.
4. The quantity of water a household uses per day (WQ) is indeed determined by various factors such as family size, distance from the water point, and water supply and management strategies. These factors can make the variable endogenous, meaning that it is influenced by other variables in the equation specified (see Equation 5). To account for endogeneity, researchers often employ instrumental variable approaches, such as using distance to the water point as an instrumental variable, to obtain unbiased estimates of the effects of other variables on water quantity. This is not taken care of in the article.
5. Energy security (ES) can be influenced by the number of livestock households own, as livestock rearing may require additional energy inputs for housing, feeding, and transportation. Education status can also affect energy security as it might be associated with income levels and access to modern energy sources. However, it is important to note that the specific mechanisms through which livestock ownership and education status affect energy security may vary and require further investigation. The authors need to take this issue into account.
6. Regarding the dummy variables WS, ES, and FS, without information on the cut-off values, it is challenging to interpret their exact meaning. Besides, while these variables are related, they are included in the household well-being equation (Equation 12) as separate indicators including almost similar variables affecting them. To address concerns about endogeneity and explore relationships among these variables, I suggest structural equation modeling, as it allows for the simultaneous examination of relationships between WS, ES and FS while estimating well-being. In fact, well-being is not measured in the article, and I wonder how the authors estimated it without measuring it.
