# Supplementary material for: Effects of water, energy, and food security on household well-being
Source: PLoS One. 2024 Jul 11;19(7):e0307017. doi: 10.1371/journal.pone.0307017 (PMC11238993; doi:10.1371/journal.pone.0307017)
Supplement: Attachment — Submitted filename: RESPONSE TO REVIEWERS COMMENTS.docx [file pone.0307017.s004.docx]

**AUTHORS’ RESPONSE TO REVIEWERS' COMMENTS**

We appreciate the editors’ and the reviewers' constructive comments that have further improved this manuscript. Based on your comments and other suggestions made, we provide the following responses.

**Reviewers' comments**

General comment:

**In a positive note, the authors have raised an important issue of concern in Africa, well-being affected by energy, food, and water security. These are issues of huge policy relevance.**

**Response**: We deeply appreciate the reviewer's positive assessment of our work and their recognition of the importance of the issues we address. It's encouraging to know that our research resonates with the significance of challenges related to energy, food, and water security in Ghana.

**Comment	1#:**	WMS (Water supply systems) can indeed vary by household, even within the same locality. Factors such as infrastructure condition, household connections, and water usage patterns can lead to variations in water supply. However, in one village, water could be centrally managed, making this variable to be rather fixed for all households residing in that village. To deal with the fixed effects nature of the variable in a village where several sample households might have been considered for the analysis, there is a need to employ such statistical techniques as village fixed-effects regression to account for village-specific differences while estimating the effects of water management on water security.

Response: Thank you for your insightful comment regarding the variability of water supply systems (WMS) within households and villages. We agree that factors such as infrastructure condition and household connections can lead to diverse water supply experiences, even within the same locality. We acknowledge that in some villages, water management may indeed be centrally controlled, resulting in a relatively fixed variable across households. To address the fixed effects nature of the variable, particularly in villages where multiple sample households are considered, statistical techniques such as village fixed-effects regression are often employed to account for village-specific differences. However, it's important to note that fixed effects models assume time-invariant effects (Verbeek, 2017), which may not hold in our case, given that the Ghana Living Standards Survey (GLSS) data represents a single point survey of households across the country. Despite the limitations of fixed effects models in this context, we have endeavored to capture the influence of water supply management on water security by measuring it as a dummy variable, with 1 indicating public supply management and 0 indicating private supply management. In our study, we use a binary variable to indicate whether the water supply is managed publicly or privately. While this approach does not account for the nuances of management within villages, it does allow us to evaluate the overall impact of various management systems on household water security.

Comment 2#: Remittance and employment status can affect water security in multiple ways. Remittances can increase a household’s purchasing power, enabling them to invest in better water infrastructure or access alternative water sources. Similarly, employment status can influence water security as it affects a household’s income stability and ability to afford reliable water services. Nevertheless, the authors disregard this reality in their estimation.

Response: We appreciate the reviewer's instructive comment regarding the impact of remittance and employment status on water security, and we acknowledge the oversight in our initial estimation. Recognizing the multifaceted nature of water security, we have integrated remittance and employment status variables into our model to more accurately capture their influence.

Comment 3#: Regarding livestock ownership (LSO), it is true that households with more livestock may have less water available for other purposes. This is because livestock require water for their own needs, and households often share their water supply with the animals. As a result, the water that could have been used for domestic or other productive purposes is allocated to livestock, potentially affecting overall water availability for the household. However, this is not considered in the regression model used.

Response: We appreciate the reviewer's useful observation about how livestock ownership may affect water availability in households. Recognizing the importance of this factor, we included livestock ownership (LSO) as a variable in our regression model to better understand its impact on overall water availability and usage patterns.

Comment 4#: 4.	The quantity of water a household uses per day (WQ) is indeed determined by various factors such as family size, distance from the water point, and water supply and management strategies. These factors can make the variable endogenous, meaning that it is influenced by other variables in the equation specified (see Equation 5). To account for endogeneity, researchers often employ instrumental variable approaches, such as using distance to the water point as an instrumental variable, to obtain unbiased estimates of the effects of other variables on water quantity. This is not taken care of in the article.

Response: We appreciate the reviewer's astute observation regarding the potential endogeneity of the variable representing the quantity of water a household uses per day (WQ) in our model. Recognizing the importance of addressing this issue to ensure the validity of our estimates, we employed instrumental variable (IVPROBIT) methodology in our analysis. Incorporating instrumental variable approaches into our study, we aimed to mitigate potential endogeneity concerns and enhance the robustness of our findings. Thank you for highlighting this critical aspect.

Comment 5#: Energy security (ES) can be influenced by the number of livestock households own, as livestock rearing may require additional energy inputs for housing, feeding, and transportation. Education status can also affect energy security as it might be associated with income levels and access to modern energy sources. However, it is important to note that the specific mechanisms through which livestock ownership and education status affects energy security may vary and require further investigation. The authors need to take this issue into account.

**Response**: We appreciate the reviewer's instructive comment about the potential effects of livestock ownership and educational level on energy security. In our analysis, we did include livestock ownership and education status as variables in our model, recognizing their significance in determining household energy security.

Comment 6 #: Regarding the dummy variables WS, ES, and FS, without information on the cut-off values, it is challenging to interpret their exact meaning. Besides, while these variables are related, they are included in the household well-being equation (Equation 12) as separate indicators including almost similar variables affecting them. To address concerns about endogeneity and explore relationships among these variables, I suggest structural equation modeling, as it allows for the simultaneous examination of relationships between WS, ES and FS while estimating well-being. In fact, well-being is not measured in the article, and I wonder how the authors estimated it without measuring it.

**Response**: We appreciate the detailed feedback provided by the reviewer regarding the interpretation of dummy variables WS, ES, and FS, as well as their inclusion in the household well-being equation. We acknowledge the importance of clarity regarding the cut-off values for these variables, and we apologize for any confusion. To clarify, in our study, WS, ES, and FS scores ranged from negative to positive values, with a cut-off value of zero. Values below zero were considered indicative of least security, while values above zero were deemed highly secure. We also recognize the interconnectedness of water security, energy security, and food security at the macro level, particularly in terms of their roles in energy generation and food preservation and production. However, at the household level, we treat WS, ES, and FS as independent variables due to their distinct impacts on household well-being. Regarding the measurement of well-being, we proxy it with quintile welfare, which is derived from the Ghana Living Standards Survey (GLSS) and ranges from 1 to 5, with 1 representing lower welfare and 5 indicating higher welfare. While we acknowledge the potential benefits of using structural equation modeling (SEM) to explore relationships among these variables, we believe that our current approach provides valuable insights into the determinants of household well-being within the context of water, energy, and food security. Thank you for your thoughtful suggestions, and we have ensured this clarification of these aspects in our methodology for improved understanding.
