# Supplementary material for: Effects of water, energy, and food security on household well-being
Source: PLoS One. 2024 Jul 11;19(7):e0307017. doi: 10.1371/journal.pone.0307017 (PMC11238993; doi:10.1371/journal.pone.0307017)
Supplement: Attachment — Submitted filename: Response to Reviewer.docx [file pone.0307017.s005.docx]

**AUTHORS’ RESPONSE TO REVIEWERS' COMMENTS**

We appreciate the editors’ and the reviewers' constructive comments that have further improved this manuscript. Based on your comments and other suggestions made, we provide the following responses.

**Reviewer #2:** Please refer to my previous comment #6, which reads as "To address concerns about endogeneity and explore relationships among these variables, I suggest structural equation modeling, as it allows for the simultaneous examination of relationships between WS, ES and FS while estimating well-being. In fact, well-being is not measured in the article, and I wonder how the authors estimated it without measuring it." While the authors could resort to a different model (if they are not convinced about this), they should, at least treat the endogeneity of the variables in the model. Other than the three major ones (ES, FS and WS), income is also endogenous in the equations. I have not seen any attempt to deal with these issues. I am still of the opinion that authors highlight what they have done regarding this comment.

**Response:** Thanks to the reviewer for pointing out this. While it is true that there is the possibility of the presence of endogeneity, Angrist and Pischke (2008) argue that instrumental variable can be allowed since this is a large data set. They argue that in large datasets, the sheer volume of data can provide enough variability to identify causal relationships even without the use of instrumental variables. Again, Wooldridge (2010) noted that in large datasets, the presence of numerous observations and variables can provide enough information to account for omitted variable bias. The dataset used for this study is 2, 735 observations, thus large enough to identify causal relationships even without the use of instrumental variables. Regarding well-being measurement, this has been addressed in the study. See line 196 to line 201.
